# Mechanical programming of arterial smooth muscle cells in health and ageing

**DOI:** 10.1007/s12551-021-00833-6

**Published:** 2021-08-30

**Authors:** Robert T. Johnson, Reesha Solanki, Derek T. Warren

**Affiliations:** School of Pharmacy, University of East Anglia, Norwich, NR4 7TJ UK

**Keywords:** Mechanotransduction, Cytoskeleton, Arterial compliance, Arterial smooth muscle cell

## Abstract

Arterial smooth muscle cells (ASMCs), the predominant cell type within the arterial wall, detect and respond to external mechanical forces. These forces can be derived from blood flow (i.e. pressure and stretch) or from the supporting extracellular matrix (i.e. stiffness and topography). The healthy arterial wall is elastic, allowing the artery to change shape in response to changes in blood pressure, a property known as arterial compliance. As we age, the mechanical forces applied to ASMCs change; blood pressure and arterial wall rigidity increase and result in a reduction in arterial compliance. These changes in mechanical environment enhance ASMC contractility and promote disease-associated changes in ASMC phenotype. For mechanical stimuli to programme ASMCs, forces must influence the cell’s load-bearing apparatus, the cytoskeleton. Comprised of an interconnected network of actin filaments, microtubules and intermediate filaments, each cytoskeletal component has distinct mechanical properties that enable ASMCs to respond to changes within the mechanical environment whilst maintaining cell integrity. In this review, we discuss how mechanically driven cytoskeletal reorganisation programmes ASMC function and phenotypic switching.

## Introduction

The cellular components of our blood vessels are subjected to numerous mechanical forces, none more so than those of the aorta, the largest vessel in our body. Cells have evolved to not only withstand the stresses and strains of these forces, but also to adapt their structure and function in response to them. Large-elastic arteries, including the aorta, possess the ability to change shape in response to changes in blood pressure, a property known as arterial compliance. Maintenance of arterial compliance is essential for healthy ageing, with decreased compliance and vascular stiffening being major risk factors in the development of cardiovascular disease (CVD). Vascular stiffening has two main effects: (1) it will increase pulse wave velocity and lead to damage of delicate microvascular vessels, and (2) it places increased workload on the heart, increasing the risk of heart failure (Glasser et al. 1997; Mitchell et al. 2010).

**Fig. 1:**
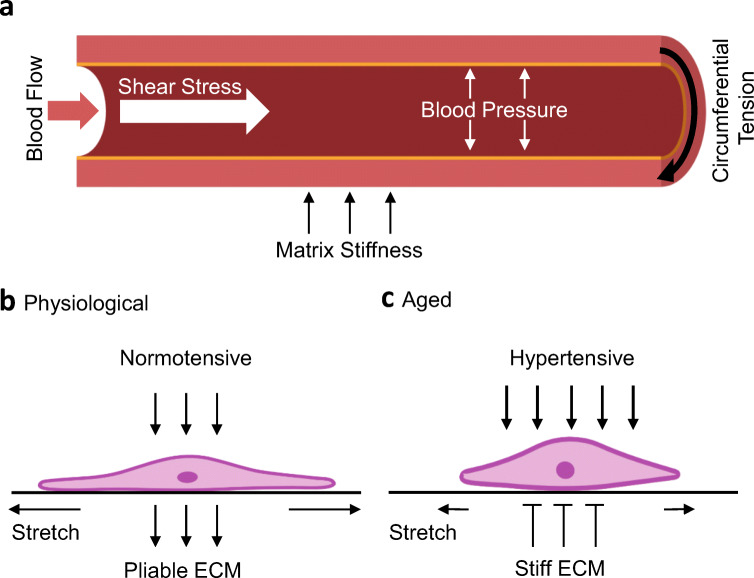
Mechanical forces acting on ASMCs change with age. **a** Mechanical forces regulating arterial function are derived from both blood flow (shear stress and pressure) and the supporting extracellular matrix (stiffness, topography and curvature). Mechanical forces, including blood pressure and matrix rigidity, regulate ASMC function. **b** Under physiological conditions, the arterial wall is compliant and stretches in response to the force of blood pressure pushing against it. ASMCs respond to the combined signals of blood pressure (compressive force) and wall stretch by initiating actomyosin-driven contraction. **c** During ageing, increased blood pressure (hypertension) generates additional compressive force. Additionally, the stiffness of the arterial wall increases, which decreases compliance and reduces the ability of the arterial wall to deform. Therefore, ASMCs experience increased compressive force and decreased stretch. Age-related changes in mechanical cues enhance actomyosin-driven contractility which further decreases compliance

Arterial smooth muscle cells (ASMCs) are the predominant cell type within the arterial wall. ASMC contraction is initiated by chemical, electrical and mechanical factors, and acts to reduce arterial compliance (Lincoln et al. 2001; Brozovich et al. 2016; Ahmed and Warren 2018). Mechanical forces regulating vascular function can be derived from both blood flow (pressure, tensile strain and shear stress) and the supporting extracellular matrix (ECM) (stiffness, topography and curvature) (Fig. 1a). A summary of these forces and their regulation of the endothelium has been reviewed recently (Dessalles et al. 2021). A key hallmark of vascular ageing and CVD onset is arterial stiffening (Sethi et al. 2014). ASMCs respond to increased matrix rigidity by enhancing actomyosin force production (Qiu et al. 2010; Sehgel et al. 2015). The pathways responsible for ECM rigidity-dependent actomyosin activity, and whether ASMCs generate large enough forces to contract rigid arterial walls, remain unknown.

Mechanotransduction, the ability of cells to convert mechanical stimuli into biochemical signals was traditionally the function of specialised membrane-embedded ion channels. However, it is now clear that integrins, clustered within focal adhesions (FA), can also transduce mechanical signals from the ECM (Alenghat and Ingber 2002; Ohashi et al. 2017). The ASMC contractile phenotype is found to be dependent on the expression of specific integrins (Zargham and Thibault 2006). In response to intraluminal pressure or cell adhesion–associated tension, focal adhesion kinase (FAK), a mechanosensitive component of FAs undergoes autophosphorylation (Lehoux et al. 2005; Ribeiro-Silva et al. 2021). Phosphorylated FAK activates Src and potentiates FA remodelling, via the phosphorylation of downstream signalling molecules paxillin (PXN) and CAS. Once activated, pPXN and pCAS initiate actin polymerisation through a Rac/RhoA mediated pathway (Ribeiro-Silva et al. 2021). FAs have been shown to be major regulators of ASMC contractility and tone. Inhibition of the FAK-Src pathway perturbs FA dynamics and reduces ASMC stiffness (Saphirstein et al. 2013). During ageing, Src expression decreases, impairing ASMC plasticity and reducing the ability of FA complexes to absorb hemodynamic forces (Gao et al. 2014).

Although FAs can transduce mechanical force, in order to initiate a structural or mechanical response, these signals must be propagated through the load-bearing architecture of the cell, the cytoskeleton (Alenghat and Ingber 2002; Ohashi et al. 2017). The mammalian cytoskeleton is comprised of three core components: the compression-resistant microtubule network, and the tension-bearing actin cytoskeleton and intermediate filament networks. The mechanical properties of each component work synergistically to prevent cell rupture under a range of mechanical stresses and strains (Sanghvi-Shah and Weber 2017). Of these cytoskeletal components, the role of the actin cytoskeleton in ASMC mechanotransduction is the most studied, due to both its role in actomyosin force generation and its direct association with FAs. Actin filaments bind to the cytoplasmic tail of β-integrins via a talin bridge. This interaction is transient under low strains, but as tension increases talin undergoes a conformational change that enables the actin-talin linkage to be reinforced through the binding of vinculin (Lacolley et al. 2018). As matrix rigidity increases during ageing, greater strain is placed onto FAs and vinculin is recruited to stabilise the FA-cytoskeleton linkage (Lacolley et al. 2018). Further discussion on the role of FA complexes in regulating ASMC function, phenotype and ECM organisation can be found in recent reviews (Ohanian et al. 2015; Lacolley et al. 2018; Ribeiro-Silva et al. 2021).

In this review, we explore how the mechanical forces imposed upon the arterial wall are transduced through the ASMC cytoskeleton, thereby regulating ASMC contractility and phenotype. Although smooth muscle cells are constituents of both the arterial and venous vascular wall, compositional and functional differences between these vascular beds alter their behaviour and response to mechanical stimuli (Wadey et al. 2018). As such, this review will solely focus on the role of ASMCs. Additionally, there are differences between ASMCs depending on their vessel of origin, most notably between large-elastic and small-muscular arteries (Majesky 2007; Chi et al. 2007; Owens et al. 2010). Whilst not the main focus of this review, we will indicate any vessel-specific differences that alter ASMC mechanoresponse. Finally, we will discuss how the ASMC cytoskeleton adapts to a changing mechanical environment, focusing particularly on changes associated with ageing. These being increased blood pressure and enhanced aortic stiffness, early biomarkers of CVD.

## Aortic compliance and age-associated changes to mechanical cues

In healthy arteries, blood pressure pushes against the arterial wall, causing it to stretch. ASMCs respond to both the compressive force of blood pressure and the stretch of the arterial wall by initiating actomyosin-driven contraction (Ye et al. 2014) (Fig. 1b). During ageing, these mechanical cues change dramatically. Firstly, increased blood pressure results in enhanced blood-derived forces. Secondly, the rigidity of the arterial wall increases, as a consequence of ECM remodelling. Over time, elastin, the elastic component of the arterial wall, degrades and is replaced by collagen-I, providing increased tensile strength to the arterial wall (Tsamis et al. 2013). As the rigidity of the ECM increases, arterial compliance decreases, meaning the arterial wall is now resisting the blood-derived forces (Ahmed and Warren 2018). Under these altered mechanical cues, ASMCs experience decreased stretch but increased compressive forces. In response, ASMCs generate enhanced actomyosin-derived contractile forces and further decrease arterial compliance (Ye et al. 2014) (Fig. 1c). Furthermore, ageing and hypertension lead to the accumulation of ASMC DNA damage (Ragnauth et al. 2010; Meloche et al. 2014). DNA damage accumulation promotes ASMC dedifferentiation and senescence, and has been linked to the formation and subsequent severity of atherosclerotic lesions (Gray et al. 2015).

## ECM topology, ASMC morphology and function

**Fig. 2:**
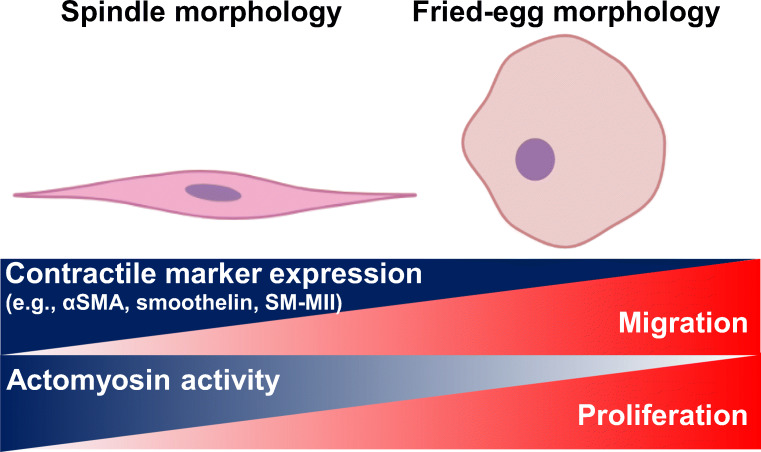
Changes in morphology allow mechanical programming of ASMC phenotype. ASMCs are not terminally differentiated and possess the ability to switch between contractile and proliferative phenotypes. In vivo, quiescent ASMCs adopt a contractile, spindle-shaped morphology and express smooth muscle contractile markers, including α-smooth muscle actin (αSMA), smoothelin and smooth muscle myosin II (SM-MII). ASMCs dedifferentiate into a proliferative phenotype when grown in culture or upon mechanical or biochemical stimulation. Proliferative ASMCs downregulate contractile marker proteins and lose their spindle shape, adopting a morphology akin to a fried egg. Cytoskeletal reorganisation within proliferative ASMCs reduces actomyosin activity and promotes cell migration and proliferation

ASMCs are not terminally differentiated and possess the ability to switch between contractile and proliferative phenotypes (Ahmed and Warren 2018). Despite much research, our understanding of this phenotypic switching remains poor. In the arterial wall, ASMCs exist in a quiescent/contractile phenotype and adopt a spindle-like morphology (Alford et al. 2011). Traditionally, ASMCs have been cultured on plastic and glass, which are around a thousand times stiffer than the arterial wall. However, when ASMCs are isolated and expanded, they exist in a proliferative phenotype and adopt a spread, fried egg-like morphology (Fig. 2). Whilst serum withdrawal promotes ASMCs to adopt a quiescent phenotype, this does not fully recapitulate the contractile phenotype displayed in vivo. Notably, expression of myosin heavy chain (MYH11) isoforms SM1 and SM2 remain downregulated in cultured ASMCs (Babij et al. 1992; Han et al. 2006). Additionally, cultured ASMCs are typically assayed as individual cells, not as a functional, multi-layered unit. This means that the importance of communication between neighbouring ASMC and endothelial cells can be overlooked. However, the transmission of signals between ASMCs via connexin (CX) gap junctions contributes to ASMC plasticity, with the vaso-protective function of CX40 promoting a contractile phenotype and upregulation of CX43 associated with ASMC dedifferentiation (Myasoedova et al. 2020).

Multiple soluble factors and ECM rigidity have been shown to regulate phenotypic switching and proliferation (Ahmed and Warren 2018; Afewerki et al. 2019). More recent studies have used ECM topological control to define ASMC shape and orientation (Agrawal et al. 2015). Importantly, these studies suggest that ASMC function and potentially phenotype are tightly coupled to morphology. Adhesion, proliferation, expression of smooth muscle myosin and myogenesis have all been linked to ASMC morphology (Thakar et al. 2009; Alford et al. 2011; Williams et al. 2011; Chaterji et al. 2014; Zhang et al. 2016). These findings suggest that when culturing ASMCs, ECM stiffness and topological control are important considerations. Given the current speed of technological advancement, we are heading towards systems that will enable a more complete understanding of ASMC phenotypic control.

Despite the recent advancements in our understanding of the mechanical and organisational regulation of ASMC differentiation and function, we still lack a clear understanding of how these cues are transduced. The ASMC cytoskeleton potentially plays a pivotal role in resisting and responding to mechanical cues. The cytoskeleton is also critical in defining cell morphology. Therefore, we will next discuss how the cytoskeleton regulates ASMC mechanotransduction.

## Actin filaments—more than just a contractile apparatus

The most studied component of the ASMC cytoskeleton is actin filaments. Actin accounts for around 20% of VSMC protein content and is also a key regulator of actomyosin-driven ASMC contractility (Kim et al. 2008). However, actomyosin-driven contractility is not the only mechanosensitive role of the actin cytoskeleton. Of the 6 actin isoforms, ASMCs have been shown to express α-smooth muscle actin (αSMA), β-actin (also known as β-non-muscle or β-cytoplasmic actin), γ-smooth muscle actin (γSMA) and γ-cytoplasmic actin, in order of decreasing proportion (Kim et al. 2008). Dedifferentiation of ASMCs from a contractile phenotype reduces the expression of contractile actin isoforms αSMA and γSMA (Rensen et al. 2007). Actin exists within two states, either a globular G-actin monomer or as filamentous F-actin, an elongated, polar polymer (Gunst and Zhang 2008). Transition between the two states is regulated by ATP binding and hydrolysis. ATP-bound G-actin typically binds at the fast-growing barbed end of the actin filament, whilst ADP-bound monomers generally dissociate from the opposing end, causing the filament to treadmill (Wear et al. 2000). ASMCs generate contractile force through the ATP-driven association between actin filaments and myosin II. The mechanism through which actomyosin complexes generate force has been extensively reviewed previously (Gunst and Zhang 2008; Tojkander et al. 2012; Yamin and Morgan 2012). In response to blood-derived compression and stretch, ASMCs generate actomyosin-driven contractility through the actions of two interlinked pathways: the calcium-dependent and the Rho-ROCK pathway (Ahmed and Warren 2018).

Upon mechanical loading, ASMCs reorganise their actin cytoskeleton via a two-step response. Seconds after a force is applied, actin filaments are stretched and align along the direction of the force (Li et al. 2020). A few minutes later, actin filaments become stabilised through the ATP-dependent process of αSMA crosslinking. This enables myosin to interact with the actin bundle, generating actomyosin-driven contractile forces and providing a site for further actin filament recruitment (Li et al. 2020). This demonstrates the dynamic nature of the actin cytoskeleton and its responsiveness to external mechanical stimuli. As we age, the rigidity of the aortic wall increases (Qiu et al. 2010). ASMCs sense increased matrix stiffness and remodel their actin cytoskeleton. On a collagen-I matrix, as stiffness increases, actin stress fibres align, decreasing ASMC migration speed and distance (Sanyour et al. 2019; Rickel et al. 2020). Actin stress fibre alignment enhances actomyosin force generation, stiffening ASMCs and further reduces arterial compliance (Sazonova et al. 2015; Sanyour et al. 2019). However, this response is not uniform across all ECM substrates. On rigid, fibronectin-coated matrices actin stress fibre organisation decreases, and ASMC migration speed and persistence increase (Rickel et al. 2020). In addition, ASMCs undergo dedifferentiation and adopt a proliferative phenotype (Sazonova et al. 2015). This indicates that ECM composition and stiffness are both important regulators of ASMC function.

Cycle-by-cycle variations in blood pressure exert mechanical forces of variable amplitude onto ASMCs. These variations programme the contractile response of ASMCs through fluctuation-driven mechanotransduction. Isolated aortic rings placed under monotonous stretching (cycles of consistent strain and relaxation) display a reduced contractile response in contrast to those undergoing variable stretching (variable strain per cycle) which maintain a physiological level of contractility (Bartolák-Suki et al. 2015). The application of stretch triggers the actin cytoskeleton to reorganise, increasing its organisation through the alignment of actin filaments. This reorganisation enables the cell to maintain intracellular tension whilst under deformation (Bartolák-Suki et al. 2015). When exposed to variable stretch, the amount of elastic potential energy communicated to the cell is greater. This leads to an enhanced alignment of the actin cytoskeleton, with cortical actin in addition to contractile stress fibres undergoing reorganisation (Bartolák-Suki et al. 2015). Actin cytoskeleton reorganisation enhances the dispersal of tension and enables the conduction of extracellular forces onto intercellular organelle. Stretch-induced reorganisation of the actin cytoskeleton in turn leads to reorganisation of the mitochondrial network. Alignment of mitochondria enhances their efficiency, increasing ATP production and decreasing reactive oxygen species (ROS) by-production (Bartolák-Suki et al. 2015; Bartolák-Suki and Suki 2020). These effects are again enhanced in ASMCs exposed to variable as opposed to monotonous stress. Increased availability of ATP enhances actin filament polymerisation and actomyosin force generation, establishing a positive feedback loop which maintains the contractile ability of ASMCs (Bartolák-Suki et al. 2015; Bartolák-Suki and Suki 2020). ASMCs are optimally tuned to physiological levels of blood pressure variability. Pathologically high levels of variability experienced during hypertension disrupt the mitochondrial network and enhance ROS production (Bartolák-Suki and Suki 2020). Elevated ROS impedes ASMC relaxation and promotes enhanced expression of contractile proteins, prolonging the contractile tone and stiffening the cell (Bartolák-Suki and Suki 2020). However, the role of ROS in regulating ASMC phenotype is not definitive, with ROS generation shown to both promote ASMC differentiation and dedifferentiation (Pi et al. 2013; Lee et al. 2016; Montezano et al. 2018; Tóth et al. 2020).

## Mechanical remodelling of the actin cytoskeleton regulates transcription

The actin cytoskeleton has been implicated in transcriptional regulation via the myocardin-related transcription factors (MRTF). MRTF-A is sequestered in the cytoplasm via interactions with monomeric G-actin. Upon actin polymerisation, MRTF-A dissociates and translocates to the nucleus where it associates with the serum response factor (SRF) and promotes SRF-dependant transcription (Mouilleron et al. 2011). SRF activation by MRTF-A promotes the expression of smooth muscle-specific contractile proteins (Wang et al. 2003; Hinson et al. 2007). Cell morphology has also been implicated in MRTF-A nuclear localisation (O’Connor and Gomez 2013). Importantly, MRTF-A nuclear translocation is also mechanically regulated. Matrix rigidity and stretch induce actin polymerisation and promote MRTF-A nuclear accumulation (Dai et al. 2019; Montel et al. 2019). Activation of MRTF-A has also been implicated in ASMC stiffening during hypertension, further confirming the importance of this pathway (Lacolley et al. 2017).

Biomechanical stretching of ASMCs inhibits the Hippo-kinase pathway, enabling the activation and nuclear localisation of the transcriptional co-activators YAP/TAZ (Wang et al. 2018). Nuclear accumulation of YAP/TAZ promotes ASMC dedifferentiation, silencing the expression of smooth muscle contractile markers and establishing a proliferative and proinflammatory phenotype (Wang et al. 2018). Active YAP reduces contractile marker expression by disrupting the interaction between SRF and its co-activator myocardin (Xie et al. 2012). During hypertension, elevated angiotensin II signalling promotes the upregulation of YAP expression within ASMCs. Inhibition of YAP or F-actin depolymerisation alleviates hypertension-associated vascular remodelling and ASMC dysfunction (Lin et al. 2018). Cell shape and mechanical tension transmitted through the actin cytoskeleton have been shown to regulate nuclear YAP/TAZ accumulation in a variety of cell types (Dupont et al. 2011). Given that the ASMC actin cytoskeleton undergoes reorganisation in response to changes in the mechanical environment, it stands to reason that these changes additionally regulate ASMC activity and phenotype through the regulation of YAP/TAZ.

**Fig. 3:**
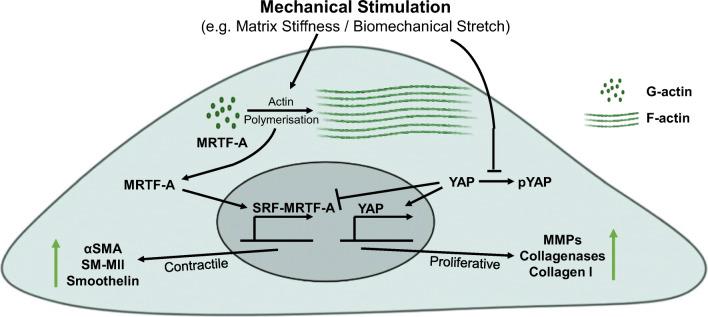
Transcriptional regulation of ASMC phenotype. Mechanical stimulation (e.g. matrix stiffness or biomechanical stretch) promotes actin filament polymerisation, depleting the cytosolic pool of G-actin and releasing myocardin-related transcription factor A (MRTF-A). MRTF-A translocates to the nucleus where it interacts with serum response factor (SRF) and promotes ASMC contractile differentiation via the expression of contractile markers including α-smooth muscle actin (αSMA), smoothelin and smooth muscle myosin II (SM-MII). Conversely, mechanical stimulation also activates the transcriptional co-activator YAP through inhibiting its phosphorylation and cytoplasmic retention. Active YAP translocates to the nucleus and promotes ASMC dedifferentiation into a proliferative phenotype, promoting the expression of genes associated with extracellular matrix remodelling including matrix metalloproteases (MMPs), collagenases and collagen-I. Furthermore, active YAP disrupts the SRF-MRTF-A interaction, preventing the expression of smooth muscle contractile markers

It is clear that ASMC phenotype is regulated by mechanical cues and the actin cytoskeleton. MRTF-A and YAP/TAZ are critical components of this regulation (Fig. 3). YAP/TAZ promotes ASMC dedifferentiation, whilst MRTF-A generally promotes the ASMC contractile phenotype, although one study has shown that following vascular injury MRTF-A upregulation contributes to enhanced ASMC proliferation (Minami et al. 2012). However, both pathways are regulated by similar mechanical cues. We lack an understanding of the balance between these conflicting pathways. For example, is there a mechanical threshold that differentiates between MRTF-A and YAP/TAZ nuclear accumulation? Is it the same for ASMCs from different vascular beds/embryonic origins? Further research is needed to dissect the precise mechanisms and thresholds regulating these pathways.

## Microtubules—compression-resistant struts

In response to exogenous force, microtubules are proposed to be compression-bearing, providing a resistive force against deformation (Brangwynne et al. 2006). The cellular tensegrity model proposes that interactions between actin filaments, microtubules and the ECM regulate cell stiffness, shape and deformability (Stamenović 2005). Microtubules and the ECM balance pre-existing contractile stress generated by actin filaments, providing shape and stability to the cell (Stamenović 2005). Microtubules are hollow, tube-like structures comprised of a helical array of polar protofilaments, formed from the polymerisation of α/β-tubulin heterodimers. Existing in a state of dynamic instability, microtubules constantly go through phases of growth and shrinkage (Nogales 2001). This intrinsic property enables microtubules to rapidly respond to the changing requirements of the cell and regulate processes including cell division, migration and intracellular transport.

Interest in the ability of microtubules to regulate ASMC force generation peaked around the turn of the century. Microtubule depolymerisation increased ASMC contractility and isometric force production (Leite and Webb 1998). This finding held true regardless of the pharmacological method of microtubule depolymerisation, or the mechanical or agonistic approach used to induce contractility, although some vessel-specific variations were reported (Sheridan et al. 1996; Platts et al. 1999, 2002; Paul et al. 2000; Zhang et al. 2000). This microtubule depolymerisation-dependent-enhanced force generation could be blocked by pre-treating ASMCs with the microtubule stabilising agent paclitaxel (brand name Taxol) (Paul et al. 2000). However, paclitaxel treatment alone has no effect on ASMC contractility (Zhang et al. 2000). Actomyosin-driven ASMC contractility is regulated by Ca^2+^-dependent and -independent pathways, both of which are upregulated following microtubule destabilisation (Sheridan et al. 1996). Increased intracellular Ca^2+^ levels are detected in pulmonary and coronary artery rings following microtubule depolymerisation (Paul et al. 2000). Meanwhile, enhanced contractility within muscular arterioles can be blocked by inhibiting the Rho-kinase pathway, which promotes actomyosin activity through the phosphorylation of the myosin light chain (Platts et al. 2002). Microtubules regulate Rho-kinase activity through the RhoA guanine nucleotide exchange factor (GEF)-H1, which is usually microtubule bound but is released upon microtubule depolymerisation (Krendel et al. 2002). Release of GEF-H1 activates RhoA and enhances actin stress fibre formation and actomyosin-driven contractility (Krendel et al. 2002). Inhibition of Rho-kinase blocked increased actin stress fibre and focal adhesion formation in 3T3 fibroblasts following microtubule depolymerisation (Liu et al. 1998). In addition to enhancing force generation, microtubule disruption (depolymerisation or stabilisation) also reduced the ability of vessels to vasodilate. Impaired vasodilation occurred through both endothelial-dependent (reduced nitric oxide production) and endothelial-independent mechanisms (Leite and Webb 1998; Platts et al. 2002; Hemmer et al. 2009).

Despite microtubule depolymerisation resulting in enhanced force generation and impaired vasodilation, early studies found no increase in arterial stiffness, leading researchers to believe that microtubules regulated ASMC contractility solely through biochemical pathways (Paul et al. 2000). However, the tensegrity model of microtubule compression predicts that it is the resting equilibrium of load spread between microtubules, actin filaments and the ECM that will determine how a cell responds to microtubule disruption (Stamenović 2005). In contractile ASMCs, microtubules align roughly parallel to actin filaments in a fibrillar pattern (Zhang et al. 2000). Under low strains, microtubule disruption has no effect on ASMC stiffness, but as strain increases, microtubules account for approximately 30% of ASMC tensile stiffness (Nagayama and Matsumoto 2008). Therefore, microtubules act to resist the intercellular tension generated through actomyosin activity, which would otherwise compress intracellular compartments. Microtubule depolymerisation lowers the mechanical resistance of the cell and allows for enhanced force generation following increased ASMC loading (Zhang et al. 2000; Nagayama and Matsumoto 2008). Surprisingly, microtubule stabilisation via paclitaxel treatment has no effect on ASMC contractility, despite increasing microtubule density by 30%. This suggests that the pre-existing microtubule network within quiescent, contractile ASMCs is set up to provide the maximal compressive resistance available to the cell, as a counterbalance to actomyosin activity (Zhang et al. 2000).

In addition to resisting compression, the microtubule network has also been shown to regulate ASMC phenotype. Microtubule destabilisation leads to increased CTGF (connective tissue growth factor) and PAI-1 (plasminogen activator inhibitor-1) expression, a response that can be inhibited by pre-treating ASMCs with a microtubule stabiliser (Samarakoon et al. 2009). Both CTGF and PAI-1 are known promoters of vascular fibrosis, with their expression leading to increased MMP (matrix metalloproteinase) activity and enhanced collagen and fibronectin secretion (Lan et al. 2013). Vascular fibrosis results in arterial stiffening. The effect of arterial matrix stiffness on ASMC microtubule dynamics is currently unknown; however, it has been shown in breast cancer lines that matrix stiffness can promote both microtubule de/stabilisation (Heck et al. 2012; Torrino et al. 2021). ASMC calcification, another CVD biomarker, has been associated with decreased microtubule stability, with microtubule stabilising agents able to impair the onset of phosphate-induced ASMC calcification (Lee et al. 2014). Therapeutic regulation of the microtubule cytoskeleton has proven effective in combatting angioplasty-induced restenosis, with clinical trials identifying neointimal ASMC accumulation can be prevented using the microtubule stabiliser paclitaxel (Sollott et al. 1995; Stone et al. 2004; Gershlick et al. 2004).

Similar to actin filaments, the microtubule network can be reorganised through the application of stress. Variable stress, as opposed to monotonous stress, contributes to a greater organisation of the network (Bartolák-Suki et al. 2015). Microtubules facilitate the intracellular transport of mitochondria; therefore, a more aligned microtubule cytoskeleton enhances the organisation of the mitochondrial network and increases the production of ATP (Bartolák-Suki et al. 2015). Furthermore, under variable stretch, VSMCs upregulate mitochondrial transport pathways, with greater mitochondrial-microtubule association detected compared to cells under monotonous or no stretch. As such, destabilising the microtubule network decreases ATP production, increases ROS release and impairs VSMC contractility (Samarakoon et al. 2009; Bartolák-Suki et al. 2015). This finding contrasts those reported earlier.

## Intermediate filaments secure the mechanical integrity of the cell

It has long been known that intermediate filaments provide cells with an ability to withstand tension and provide mechanical integrity. However, of the three main cytoskeletal components, their role in mechanotransduction provides an under-investigated area, with the majority of studies focusing on the function of actin filaments (Sanghvi-Shah and Weber 2017). Of the five cytoplasmic families of intermediate filaments, ASMCs primarily express the Type III proteins vimentin and desmin. Vimentin expression is ubiquitous, whilst desmin is primarily expressed by ASMCs within small-muscular arteries (Johansson et al. 1997; Wede et al. 2002). Additionally, sporadic expression of cytokeratin in lumen proximal ASMCs have been observed and is hypothesised to occur as cells dedifferentiate into a proliferative phenotype (Johansson et al. 1997). Unlike actin filaments and microtubules, intermediate filament polymerisation is nonpolar and occurs in the absence of enzymatic regulation. Vimentin monomers interact to form parallel dimers, which in turn associate into antiparallel staggered tetramers. These tetramers form the structural unit of vimentin polymerisation, where 8 tetramers assemble head to tail into a sheet that subsequently compacts into the rope-like structure of vimentin intermediate filaments (VIFs) (Chang and Goldman 2004).

The classical representation of VIFs is a dense, cage-like network that encompasses the nucleus, with additional filaments radiating out towards the cell periphery (Murray et al. 2014). In this representation, only a very small pool of soluble vimentin exists within the cytoplasm, unlike those of G-actin and tubulin dimers. However, these studies were performed on glass or plastic. Cells grown on substrates mimicking physiologically relevant stiffness yield far less vimentin within insoluble cytoskeletal structures (Murray et al. 2014). On the softest matrices, vimentin still forms a cage-like network around the nucleus but fails to radiate outwards. As matrix stiffness increases, VIFs expand further into the cytoplasmic region. This indicates that, much like actin filaments, the polymerisation of VIFs is dependent on the mechanical environment (Murray et al. 2014). Of the three cytoskeletal components, intermediate filaments are the most resistant to strain. Where actin filaments yield under 20% and microtubules 60% strain, VIFs can withstand more than 80% strain (Janmey et al. 1991). Vimentin displays unusual viscoelastic properties. It is less rigid under low strain but hardens as strain increases, thereby preventing VIFs from fracturing (Janmey et al. 1991). The ability of VIFs to maintain their integrity under strains where actin filaments and microtubules yield provides a mechanism for cells to respond to deformation. Stabilised VIFs transmit localised stress/strain throughout the cell, dissipating force and maintaining cell integrity (Janmey et al. 1991; Hu et al. 2019).

The role of vimentin in ASMC mechanotransduction has been investigated using the global vimentin knockout (KO) mouse (Langlois et al. 2017). A caveat to this approach is the dual expression of vimentin within endothelial cells and ASMCs that convolutes the ability to trace the biological significance of vimentin to a particular cell type. Loss of vimentin altered expression of ECM components within the subendothelial basement membrane, with increased expression of fibronectin, laminin and collagen IV (Langlois et al. 2017). Whilst these ECM components are not typically associated with increased vascular stiffness, their overexpression creates a thicker, denser ECM environment that displays an altered topography. In response, ASMCs lose their spindle-shaped lamellar organisation and decrease expression of contractile markers (Langlois et al. 2017; van Engeland et al. 2019). Phenylephrine-induced contractility increased and endothelium-dependent vasodilation decreased, leading to an overall increase in arterial stiffness (Langlois et al. 2017).

Global vimentin KO decreases the ability of resistance arteries to vasodilate in response to flow-derived shear stress (Henrion et al. 1997). Shear stress primarily affects the endothelium, perturbing ASMC relaxation by altering endothelial nitric oxide production (Quillon et al. 2015). However, the stress applied to the endothelium also transduces strain onto ASMCs. In response to strain, ASMCs undergo enhanced VIF polymerisation, increasing both their ability to withstand tension and their interaction with Jagged-1, in turn enhancing the transactivation of Notch-3 (van Engeland et al. 2019). ASMCs lacking Notch-3 promote arterial stiffening through the combined effects of enhanced pressure-induced myogenic tone and reduced flow-driven vasodilation. Furthermore, the absence of Notch-3 resulted in abnormal cytoskeletal rearrangements that correlated with altered ASMC morphology and organisation, including ASMC detachment from the overlying endothelium (Ruchoux et al. 2003; Dubroca et al. 2005). Desmin intermediate filaments are another cytoskeletal component whose role in mechanotransduction warrants further investigation. Loss of desmin reduces the circumferential tension and sustains the contractile phase within microarterial resistance vessels (Wede et al. 2002). Furthermore, ASMC desmin expression is regulated by matrix stiffness and topography, with enhanced stiffness or reduced patterning decreasing desmin expression (Chaterji et al. 2014). Intermediate filaments are clearly important for ASMC response to mechanical cues. More research is needed to define how mechanical cues in health and disease regulate this filamentous system.

## Conclusions and perspectives

VSMC differentiation and function are highly complex and intricate processes that incorporate both mechanical and biochemical components. At the heart of these processes are the cytoskeletal networks that are critical determinants of ASMC fate. Where traditional, well-defined roles for cytoskeletal components existed, it is now becoming clear that the cytoskeleton adapts as necessary following homeostatic force disruption. Emerging evidence suggests that both external and internal cues drive the mechanical programming of ASMC phenotype and function. This implies that the mechanical environment is a major determinant of ASMC fate. However, key unanswered questions remain, including: (1) the interplay between the different cytoskeletal systems in ASMC function; (2) identification of the differential mechanisms and mechanical thresholds that regulate these responses; and (3) do ASMCs from different arteries display similar response to mechanical loading? ASMC morphology and cytoskeletal organisation are key contributors to ASMC phenotype. Therefore, the development and use of tools to better model in vivo organisation and mechanical environments are essential and likely to yield novel mechanistic information that better describes ASMC function.

Whilst it is acknowledged that the mechanical forces acting on the vascular system are intertwined, when studying their effects, we typically focus on a stimulus in isolation. This has enabled the technologies required to model mechanical cues such as matrix stiffness, biomechanical stretch, flow and hydrostatic pressure to be developed. However, we lack a key understanding of the bigger picture. For example, how do ASMCs respond to the summation of these forces? In vivo, ASMCs exist in a 3-dimentional environment, yet due to technological limitations the majority of studies described in this review were performed on 2-dimentional substrates. The development of 3-dimentional scaffolds of tuneable stiffness that can be subjected to compressive forces will drive the next phase of our understanding of ASMC plasticity and function. Furthermore, ASMCs are not the only cell type within the aortic wall. Modifying existing technologies to enable the effects of mechanical cues on ASMC-endothelial cell co-cultures is essential. Combining such advancements in modelling with techniques such as bulk or single-cell RNA sequencing would enable a more thorough understanding of how ASMCs respond to mechanical stimuli. Additionally, it would help explain how mechanical insults modulate the transcriptional regulation of ASMC differentiation. These techniques have recently shown that genes associated with actin filament reorganisation become upregulated in ASMCs during ageing or disease but involvement of the mechanical landscape remains unknown (Dobnikar et al. 2018; Gao et al. 2020; Conklin et al. 2021).

## Data Availability

Not applicable.
